# Ultrasound-guided platelet-rich plasma versus corticosteroid injection for supraspinatus tendinopathy: a randomized comparative study

**DOI:** 10.1186/s12891-026-09679-z

**Published:** 2026-03-09

**Authors:** Douquan Huang, Yingfeng Ning, Xingfa Yao, Xianhua Luo, Mingjian Wang, Shiqian Zhou, Tieliang Tian, Yanli Lin

**Affiliations:** Fujian University of Traditional Chinese Medicine Affiliated Nanping People’s Hospital, Nanping, Fujian 353000 P.R. China

**Keywords:** Supraspinatus tendinopathy, Platelet-rich plasma(PRP), Local injection, Clinical efficacy

## Abstract

This study demonstrates that ultrasound-guided injection of platelet-rich plasma (PRP) is significantly effective in treating supraspinatus tendinopathy, providing higher evidence-based medical support and data.

**Methods**

A total of 60 patients meeting inclusion and exclusion criteria were selected from the Department of Orthopedics at Nanping People's Hospital, either as inpatients or outpatients, between September 1, 2021, and December 31, 2023. Patients were randomly allocated in a 1:1 ratio to either Group A or Group B. The randomization sequence was generated by a research assistant not involved in patient recruitment or assessment, using a computer-generated random number table. Group assignments were sealed in sequentially numbered, opaque envelopes. Upon a patient's enrollment, the next available envelope in the sequence was opened by the treating physician to reveal the group assignment, thereby ensuring allocation concealment.The Group A received ultrasound-guided local PRP injections, while the Group B underwent ultrasound-guided local corticosteroid injections, with both groups having a treatment duration of 2 weeks. Pain scores (using the Visual Analogue Scale, VAS), shoulder function scores (Constant score), and tendon thickness were compared at baseline,1 week,1 month,and 3 months post-treatment to evaluate the clinical efficacy of the treatment for supraspinatus tendinopathy.

**Results**

There was no statistically significant difference in VAS scores between the two groups at baseline and at 2 weeks post-treatment (*P*>0.05); however, at 1 week post-treatment, the VAS score for the Group A was higher than that of the Group B, while at 1 month and 3 months post-treatment, the VAS scores for the Group A were lower than those of the Group B, with statistical significance (*P*<0.05). For the Constant score, there were no statistically significant differences between the two groups at baseline, at 1 week, or at 2 weeks post-treatment (*P*>0.05). However, at 1 month and 3 months post-treatment, the Constant scores for the Group A were higher than those of the Group B, with statistical significance (*P*<0.05). At 3 months post-treatment, the tendon thickness in the Group A was lower than that in the Group B, with statistical significance (*P*<0.05).

**Conclusion**

Ultrasound-guided PRP injection showed superior outcomes compared to corticosteroid injection in terms of pain relief, functional improvement, and tendon thickness reduction at medium-term follow-up, though these findings require confirmation through more comprehensive imaging assessment and controlled trials.

Supraspinatus tendinopathy is a common shoulder condition that primarily affects the muscles and tendons surrounding the shoulder joint. The supraspinatus is part of the rotator cuff muscle group, located at the top of the shoulder, responsible for shoulder abduction and joint stabilization [1]. In daily life, the supraspinatus often endures significant mechanical loads, particularly during high-intensity activities or repetitive motions, making its tendon susceptible to injury and inflammation. Supraspinatus tendinopathy typically presents as shoulder pain, limited range of motion, and functional impairment [2]. Imaging examinations, such as ultrasound and MRI, can clearly display the extent of tendon damage, assisting physicians in making accurate diagnoses [3–5]. Currently, the clinical treatment principles for supraspinatus tendinopathy focus on rapidly alleviating pain and reducing inflammatory responses. Platelet-rich plasma (PRP) promotes the repair and regeneration of various tissues, including tendons, ligaments, and cartilage, and offers significant efficacy, ease of use, and minimal side effects [6]. In the context of tendon healing, PRP has been shown to be beneficial. For instance, studies have demonstrated that PRP can enhance tendon cell biology by increasing cell viability and promoting the expression of collagen, which is vital for tendon repair [7]. Additionally, PRP has been used in combination with other biological agents, such as mesenchymal stem cells (MSCs), to further augment tendon healing. This combination therapy has been reported to synergistically promote tendon-bone healing, particularly in rotator cuff injuries, by enhancing tissue regeneration and biomechanical properties [8].When it comes to cartilage regeneration, PRP has been explored as a promising therapeutic approach. It has been used in various forms, such as in combination with hydrogels, to improve the regeneration of articular cartilage. For example, PRP-loaded hydrogels have been shown to enhance hyaline cartilage regeneration by upregulating key chondrogenic markers and providing a conducive environment for chondrocyte proliferation and maturation [9]. Moreover, PRP has been combined with injectable hyaluronic acid hydrogels to facilitate cartilage healing in animal models, demonstrating improved cartilage repair outcomes [10].This study aims to observe the efficacy of ultrasound-guided local precise injection of PRP in treating supraspinatus tendinopathy, providing higher evidence-based medical support for the further promotion of this treatment protocol and enhancing the effectiveness of supraspinatus tendinopathy management.

## Clinical data

### General information

A total of 60 patients who met the inclusion and exclusion criteria were selected from the Department of Orthopedics at Nanping People’s Hospital between September 1, 2021, and December 31, 2023. Patients were randomly allocated in a 1:1 ratio to either Group A or Group B. The randomization sequence was generated by a research assistant not involved in patient recruitment or assessment, using a computer-generated random number table.Patients in Group A received ultrasound-guided local platelet-rich plasma (PRP) injections, while patients in Group B received ultrasound-guided local corticosteroid injections. there were 16 males and 14 females; the age range was 46 to 62 years, with an average age of (53 ± 1) years; 13 patients had left shoulder issues, and 17 had right shoulder issues; the duration of illness ranged from 16 to 27 months, with an average of (20 ± 1) months. In the Group A, there were 18 males and 12 females; the age range was 42 to 60 years, with an average age of (52 ± 1) years; 14 patients had left shoulder issues, and 16 had right shoulder issues; the duration of illness ranged from 12 to 25 months, with an average of (19 ± 1) months. There were no statistically significant differences (*P* > 0.05) in gender, age, number of affected shoulders, or duration of illness between the two groups, indicating comparability. This study was approved by Medical Ethics Committee of Nanping People’s Hospital, Fujian Province (2021RM006).

### Inclusion criteria

① Clear diagnosis of supraspinatus tendinopathy (excluding patients with severe supraspinatus tendon tears): The diagnosis of supraspinatus tendinopathy is based on a combination of clinical manifestations (e.g., a positive Jobe’s test, painful arc) and confirmation by shoulder MRI. MRI diagnostic criteria include: increased signal intensity within the substance of the supraspinatus tendon on T2-weighted images, without extension of the signal-defect to the articular surface or to the subacromial bursa. ② Age between 20 and 60 years, regardless of gender; ③ Voluntary participation in this clinical study and signing of an informed consent form.

### Exclusion criteria

① Patients with severe organic diseases of the heart, brain, kidneys, blood disorders, or those who are physically weak and unable to receive treatment; patients with malignant tumors; individuals with local skin ulceration or infection around the shoulder; individuals with severe systemic infections; ② Patients who have previously undergone surgery on the shoulder joint; ③ Patients with poor compliance, making it impossible to assess efficacy and safety; ④ Patients with bleeding tendencies or mental illnesses. ⑤Patients with imaging showing a full-thickness tear or a partial-thickness tear involving more than 50% of the tendon thickness were excluded.

## Methods

### Treatment methods

Patients in Group A received ultrasound-guided local platelet-rich plasma (PRP) injections, while patients in Group B received ultrasound-guided local corticosteroid injections.

#### Local block treatment

Ultrasound-guided local block injection therapy was performed as follows: the patient was seated with the shoulder joint abducted and hands on hips, while the orthopedic doctor positioned on the affected side marked the tender point at the greater tubercle of the humerus. The ultrasound doctor used ultrasound (MYLabTWice LA523 model, probe frequency 4–13 MHz) to scan the identified tender points and the supraspinatus tendon. The orthopedic doctor confirmed the lesion site identified on ultrasound by cross-referencing it with the patient’s baseline diagnostic MRI scan to ensure accurate targeting The local skin was routinely disinfected, and a sterile disposable needle (Haoguan Medical Equipment) was used to insert under the ultrasound probe, guided by real-time ultrasound observation, into the Inside the tendon sheath beneath the acromion, Avoid direct injection into the tendon, injecting 2 mL of the blocking agent. The local blocking agent consisted of 1 mL of 1% lidocaine hydrochloride (produced by Tianjin Jinyao Pharmaceutical) and 1 mL of compounded betamethasone injection (Diboson), diluted with saline to a total of 5 mL. All procedures were strictly conducted under sterile conditions. Treatments were administered once a week, for a total of two treatments.

#### Local platelet-rich plasma injection therapy

Ultrasound-guided local platelet-rich plasma (PRP) injection therapy was performed as follows: preparation of autologous PRP was conducted in a specialized sterilized operation room (guided by the hospital’s infection control department, equipped with medical plasma air disinfectors and other equipment) using radiation-sterilized vacuum blood collection tubes (specifications 2.7 mL, produced by Becton Dickinson Medical Devices (Shanghai), with a citric acid concentration of 0.109 mol/L, production batch number: 1033179). Each tube was labeled with the patient’s name, age, and hospitalization or outpatient number, and verification was conducted by the doctor, nurse, and patient. A total of 27 mL of blood (10 tubes) was drawn from the patient’s median cubital vein, and using a single centrifugation method, the collected blood was placed in a high-speed centrifuge (produced by JOANLAB) and centrifuged at 1800 r/min for 10 min, yielding approximately 3 mL of supernatant along with the leukocyte and platelet layer, referred to as PRP. We prepare leukocyte-rich platelet-rich plasma with approximately threefold platelet concentration.Throughout the procedure, the doctor and nurse wore gloves and adhered to strict sterile techniques. The patient was seated with the shoulder joint abducted and hands on hips, while the orthopedic doctor marked the tender point at the greater tubercle of the humerus. The ultrasound doctor used ultrasound (MYLabTWice LA523 model, probe frequency 4–13 MHz) to scan the identified tender points and the supraspinatus tendon, measuring its thickness and locating the lesion beneath the acromion (characterized by tendon thickening and uneven internal echogenicity, with energy Doppler imaging (PDI) showing increased blood flow). The orthopedic doctor confirmed the lesion site identified on ultrasound by cross-referencing it with the patient’s baseline diagnostic MRI The local skin was routinely disinfected, and a sterile disposable needle (Haoguan Medical Equipment) was used to insert under the ultrasound probe, guided by real-time ultrasound observation, into the lesion site beneath the acromion, injecting 2 mL of the patient’s autologous PRP. - All procedures were strictly conducted under sterile conditions. Treatments were administered once a week, for a total of two treatments.

### Efficacy observation

#### Assessment of shoulder joint pain severity

The visual analogue scale (VAS) was utilized to assess the severity of shoulder joint pain in patients at four time points: at baseline,1 week,1 month, and 3 months post-treatment。Pain intensity was measured numerically by drawing a 10 cm horizontal line on paper, with each centimeter representing one unit. One end of the line is marked “0,” indicating no pain, while the other end is marked “10,” indicating extreme pain. The middle section represents varying degrees of pain. Patients were instructed to mark their pain level on the line based on their self-perception. The marked position was then used to assign a score, where 0 indicates no pain, 1 to 3 indicates mild pain, 4 to 6 indicates moderate pain, and 7 to 10 indicates severe pain.

#### Shoulder joint function assessment

The Constant Shoulder Score system was employed to evaluate the comprehensive function of the shoulder joint in patients at four time points: at baseline,1 week,1 month, and 3 months post-treatment. The scoring was based on a 100-point scale, with the subjective component totaling 35 points, which includes pain severity and the impact on daily life. The objective component totals 65 points, encompassing shoulder joint range of motion and strength. Specifically, the pain section accounts for 15 points, daily activities for 20 points, shoulder joint range of motion for 40 points, and strength testing for 25 points.

#### Imaging assessment

**Fig. 1:**
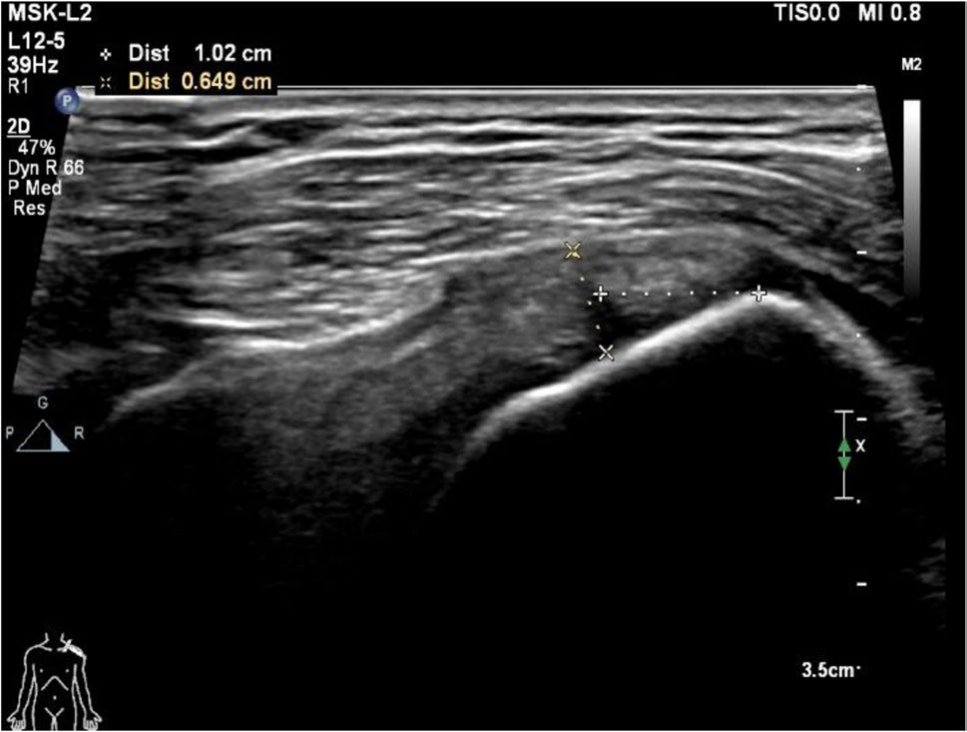
Schematic diagram of ultrasound two-dimensional image measurement. Note: The thickness of the tendon was measured at a point 10 mm away from the tendon insertion on the long axis section of the tensor muscle

The thickness of the supraspinatus tendon was measured using musculoskeletal ultrasound. All ultrasound measurements were performed by having a single, highly experienced sonographer in musculoskeletal imaging to ensure consistency. The patient was seated with the arm in the Crass position (hand behind the back) to expose the supraspinatus tendon. A long-axis view of the tendon was obtained, and the thickness was measured at a standardized point 1 cm proximal to its insertion on the greater tuberosity, perpendicular to the tendon fibers from the bursal to the articular surface. The average of three consecutive measurements was recorded for analysis(Fig. 1).Measurements were recorded before the first local injection, before the second local injection, and during the follow-up ultrasound examination three months post-treatment.To minimize measurement bias, the sonographer who performed the follow-up ultrasound examinations at 3 months was blinded to the patient’s treatment group allocation.

### Statistical methods

An intention-to-treat analysis was applied, including all randomized patients in the analysis regardless of compliance. Data analysis was performed using SPSS version 20.0. For continuous data, results are presented as mean ± standard deviation $\left(\overline{x}\:\pm\:{s}\right)$. The t-test or Wilcoxon rank-sum test was employed as appropriate. Chi-square tests were conducted for categorical data. All tests were two-tailed, with *P* < 0.05 considered statistically significant.

## Results

All 60 (100%) randomized patients completed the 3-month follow-up period, and there was no loss to follow-up.

### Comparison of VAS scores

Repeated measures ANOVA was used to compare the overall differences in VAS scores for shoulder pain at five time points: at baseline,1 week,1 month, and 3 months post-treatment, between the Group A and the Group B. The results showed F = 3.829, *P* = 0.055, indicating no statistically significant overall difference in VAS scores between the Group A and the Group B at different time points (Table 1). 

**Table 1:** Comparison of VAS between the Group A and the Group B $\left(\overline{x}\:\pm\:{s}\right)$

VAS	baseline	Treatment for 1 week	Treatment for 2 weeks	Treatment for January	Treatment for 3 months
Group A(*n* = 30)	7.33 ± 0.66	5.47 ± 0.82*	3.97 ± 0.41*#	2.90 ± 0.88*#∆	2.13 ± 0.43*#∆▯
Group B(*n* = 30)	7.60 ± 0.62	4.77 ± 0.73*	3.77 ± 0.50*#	3.57 ± 1.22*#	2.93 ± 0.52*#∆▯
F	2.592	12.238	2.822	5.853	41.76
P	0.113	0.001	0.098	0.019	< 0.001

Inter-group comparison: Compared to baseline

*P < 0.05;Compared to one week of treatment,๟*P* < 0.05;Compared to 2 weeks of treatment,△*P* < 0.05,Compared to the treatment in January,□*P* < 0.05

Between-group comparisons of VAS scores at each time point were conducted using multivariate analysis of variance. The results indicated that there was no statistically significant difference between the two groups at baseline and two weeks post-treatment (*P* > 0.05). However, the VAS scores of the Group A were higher than those of the Group B at one week post-treatment, while at one month and three months post-treatment, the VAS scores of the Group A were lower than those of the Group B, with significant differences (*P* < 0.05). The VAS score was higher in the Group A at one week, suggesting that the anti-inflammatory effect of the injection treatment was quicker and more effective in the short term. After one month, the Group A had lower scores, indicating that PRP began to gradually exert its tissue structural improvement and anti-inflammatory effects, while the Group B had only anti-inflammatory effects, which diminished over time as the anti-inflammatory medication metabolized.

Within-group comparisons of VAS scores at different time points were performed using repeated measures ANOVA, with pairwise comparisons conducted using the Bonferroni method. The results showed that for the Group A: pre-treatment > one week post-treatment > two weeks post-treatment > one month post-treatment > three months post-treatment, with statistically significant differences (*P* < 0.05); for the Group B: pre-treatment > one week post-treatment > (two weeks, one month, and three months post-treatment), and two weeks post-treatment > three months post-treatment, with statistically significant differences (*P* < 0.05). It is considered that over time, the PRP promotes the growth and structural improvement of tissue cells, with sustained anti-inflammatory and analgesic effects. In contrast, the injection medication only provides a simple anti-inflammatory and analgesic effect, and although it can reduce VAS scores over time, the extent of reduction indicates that the injection medication is less effective than PRP in medium-term pain relief.

### Comparison of constant scores

Repeated measures ANOVA was used to compare the overall differences in Constant scores between the Group A and the Group B at four time points: at baseline,1 week,1 month, and 3 months post-treatment. The results showed: F = 15.316, *P* < 0.001, indicating that the overall Constant scores of patients in the Group A were significantly higher than those in the Group B (Table 2).

**Table 2:** Comparison of CONSTANT scores between the Group A and the Group B $\left(\overline{x}\:\pm\:{s}\right)$

CONSTANT	baseline	Treatment for 1 week	Treatment for 2 weeks	Treatment for January	Treatment for 3 months
Group A(*n* = 30)	35.80 ± 6.42	66.40 ± 9.70*	72.70 ± 9.21*	80.13 ± 5.28*#∆	86.37 ± 5.99*#∆▯
Group B(*n* = 30)	37.30 ± 4.07	68.03 ± 7.17*	72.07 ± 8.89*	75.47 ± 4.66*#	75.07 ± 8.83*#
F	1.169	0.550	0.073	13.185	33.655
P	0.284	0.461	0.787	< 0.001	< 0.001

Inter-group comparison: Compared to baseline

*P < 0.05;Compared to one week of treatment,๟*P* < 0.05;Compared to 2 weeks of treatment,△*P* < 0.05,Compared to the treatment in January,□*P* < 0.05

Multivariate analysis of variance was employed to compare the Constant scores between the two groups at different time points. The results indicated that there were no statistically significant differences in Constant scores between the two groups at baseline, one week and two weeks post-treatment (*P* > 0.05), suggesting that the treatment effects on overall functional recovery were comparable during the first two weeks. However, the Constant scores for the Group A at one month and three months post-treatment were significantly higher than those of the Group B, with statistical significance (*P* < 0.05). This suggests that the pain relief effect in the Group A was superior to that of the Group B and was associated with tendon structural improvement.

Intra-group comparisons of Constant scores at different time points were conducted using repeated measures ANOVA, with pairwise comparisons performed using the Bonferroni method. The results showed: for the Group A: baseline < (one week post-treatment = two weeks post-treatment) < one month post-treatment < three months post-treatment (*P* < 0.05). For the Group B: baseline < (one week, two weeks, one month and three months post-treatment) (*P* < 0.05); one week post-treatment < (one month and three months post-treatment) (*P* < 0.05). This indicates that as time progressed, the functional indicators of the shoulder joint in both groups gradually improved, with the Group A exhibiting a greater enhancement in functional indicators compared to the Group B. This may be related to the fact that, in addition to the anti-inflammatory and analgesic effects of PRP compared to the injection, it also possesses functions related to the growth, structural improvement of cellular tissues.

### Comparison of supraspinatus tendon thickness

Repeated measures ANOVA was used to compare the overall differences in tendon thickness at four time points—at baseline,1 week,1 month, and 3 months post-treatment—between the Group A and the Group B. The results showed: F = 0.163, *P* = 0.688, indicating that there were no statistically significant differences in tendon thickness between the Group A and the Group B across different time points (Table 3).

**Table 3:** Comparison of tendon thickness between the Group A and the Group B $\left(\overline{x}\:\pm\:{s}\right)$

Tendon Thickness	baseline	Treatment for 1 week	Treatment for January	Treatment for 3 months
Group A(*n* = 30)	6.51 ± 0.99	6.45 ± 0.62	6.41 ± 0.56	6.43 ± 0.29
Group B(*n* = 30)	6.50 ± 0.63	6.43 ± 0.42	6.41 ± 0.34	6.59 ± 0.28
F	0.000	0.021	0.000	4.996
P	0.988	0.885	1.000	0.029

Inter-group comparisons of tendon thickness at each time point were performed using multivariate ANOVA. The results indicated that at 3 months post-treatment, the tendon thickness in the Group A was lower than that in the Group B, with a statistically significant difference (*P* < 0.05), suggesting that the anti-inflammatory and reparative effects of the Group A were superior to those of the Group B.

Intra-group comparisons of tendon thickness at different time points within the same group were conducted using repeated measures ANOVA, with pairwise comparisons performed using the Bonferroni method. The results revealed that there were no statistically significant differences within each group at different time points (*P* > 0.05). Notably, the tendon thickness in the Group B was higher at 3 months post-treatment compared to baseline, which may be attributed to the decline in anti-inflammatory efficacy and the impact of hormones on tendon quality after 3 months.

## Discussion

One of the primary mechanisms implicated in the development of supraspinatus tendinopathy is mechanical overload. Repetitive stress and overuse can lead to microtrauma in the tendon, resulting in a cycle of degeneration and failed healing. This mechanical loading can cause changes in the tendon structure, such as disorganization of collagen fibers and increased cellularity, which are characteristic of tendinopathy [11, 12]. Additionally, variations in acromial anatomy or altered glenohumeral kinematics can contribute to subacromial impingement, further exacerbating tendon degeneration [13].Biological factors also play a significant role in the pathogenesis of supraspinatus tendinopathy. Inflammatory mediators, such as prostaglandins and cytokines, can promote tendon degeneration and pain. For instance, PGE2, an inflammatory mediator, has been shown to decrease cell proliferation and induce osteogenic differentiation in tendon stem cells, contributing to the pathological changes observed in tendinopathy [14]. Moreover, oxidative stress has been implicated in the aberrant differentiation of tendon-derived cells, highlighting the potential for therapeutic interventions targeting oxidative pathways [15].Genetic predisposition may also influence the development of supraspinatus tendinopathy. Variations in genes related to collagen synthesis and regulation, such as COL5a1, have been associated with an increased risk of tendon abnormalities, suggesting a genetic component to the condition [16]. Furthermore, metabolic disorders, such as type 2 diabetes mellitus, have been linked to tendinopathy through alterations in metabolic pathways, including galactose metabolism, which may exacerbate tendon degeneration [17].

Platelet-rich plasma (PRP) is a biological preparation extracted from the patient’s own blood, rich in platelets and growth factors. PRP is typically obtained by centrifuging whole blood to separate it into different components, with the resulting platelet-rich plasma containing a large number of platelets, cytokines, and growth factors such as epidermal growth factor (EGF), transforming growth factor (TGF-β), and platelet-derived growth factor (PDGF). These factors aid in promoting tissue structural improvement. Additionally, PRP can reduce the factors that induce inflammatory responses in damaged tissues, alleviating inflammation and relieving pain [18]. Many clinical studies have confirmed the role of PRP in tissue damage repair and regeneration; however, there remains considerable controversy regarding its clinical efficacy. Some literature indicates that the reparative effects of PRP on tissues are related to factors such as source, extraction process, and dosage, leading to variability in clinical treatment outcomes. Based on previous studies, this treatment will utilize 2 ml of PRP to achieve optimal tendon repair effects [19].Multiple PRP (platelet-rich plasma) injections demonstrate superior therapeutic efficacy compared to single treatments for rotator cuff disorders. Studies indicate that PRP therapy can significantly alleviate pain and functional impairment in patients within a short timeframe, with sustained improvements observed during long-term follow-up [20]. Regarding multiple injection regimens, studies demonstrate that repeated PRP injections can enhance therapeutic efficacy to some extent. For instance, a study showed that three consecutive PRP injections administered two weeks apart maintained sustained symptom improvement in patients over an 18-month follow-up period [21]. It is noteworthy that while existing research has provided some support for the application of PRP, there remains a lack of clear scientific consensus regarding injection frequency. Current literature primarily focuses on the overall efficacy of PRP, with limited studies addressing specific dosing regimens (e.g., once or twice weekly). Therefore, in clinical practice, developing personalized treatment plans tailored to individual patient conditions and responses may be a more reasonable approach.The scientific rationale behind the weekly platelet-rich plasma (PRP) injection protocol has gained support from existing literature [22]. Based on our clinical practice, we have adopted a treatment plan of once every week for 2 consecutive weeks.

The present study aimed to compare the efficacy of ultrasound-guided platelet-rich plasma (PRP) injections with corticosteroid injections in the treatment of supraspinatus tendinopathy. Our results indicate that while both treatments provide significant relief from pain and improvement in shoulder function, PRP injections demonstrate superior medium-term outcomes.

In our study, both PRP and corticosteroid injections led to significant reductions in pain, as measured by the Visual Analogue Scale (VAS), and improvements in shoulder function, as assessed by the Constant Shoulder Score, across all follow-up periods. However, at the 1-month and 3-month follow-ups, the PRP group exhibited significantly greater improvements in both VAS and Constant scores compared to the corticosteroid group (*p* < 0.05). This suggests that PRP offers better sustained relief and functional recovery.

Interestingly, at the 1-week follow-up, the PRP group reported higher VAS scores, indicating more pain initially compared to the corticosteroid group. This could be attributed to the inflammatory response triggered by PRP, which is part of its mechanism to promote tissue healing. In contrast, corticosteroids provide rapid anti-inflammatory effects, leading to quicker initial pain relief but potentially less durable outcomes.

Ultrasound measurements of tendon thickness also favored the PRP group at the 3-month follow-up, with significantly reduced thickness compared to the corticosteroid group (*p* < 0.05). This finding supports the hypothesis that PRP promotes tendon healing and reduces inflammation more effectively in themedium-term.

Our findings align with several studies that have investigated the use of PRP for rotator cuff tendinopathy. For instance, Dadgostar found that both PRP and corticosteroids improved clinical symptoms in patients with rotator cuff tendinopathy, but PRP showed better results in terms of tendon structure improvement [23]. Similarly, Tanpowpong reported that PRP injections significantly reduced tear size in partial-thickness supraspinatus tendon tears, highlighting its regenerative potential [24].Our results are also consistent with a meta-analysis by Hurley et al., which concluded that PRP is more effective than corticosteroids for pain relief and functional improvement in the long term for rotator cuff injuries [25]. However, some studies have reported conflicting results. A randomized controlled trial by Kwong et al. found no significant difference between PRP and saline injections for chronic rotator cuff tendinopathy at 12 months, suggesting a potential placebo effect and highlighting the ongoing debate about PRP efficacy [26]. The variability in outcomes across studies may be attributed to differences in PRP preparation (leukocyte-rich vs. leukocyte-poor), injection volume, and patient populations. Therefore, further high-quality research is needed to standardize these variables in order to better evaluate the long-term efficacy and safety of PRP [27].

This study contributes to the existing literature by providing further evidence supporting the use of a specific, single-centrifugation, leukocyte-rich PRP preparation for supraspinatus tendinopathy. By demonstrating superior medium-term outcomes over a widely used conventional treatment (corticosteroids), our study reinforces the recommendation of PRP as a viable and potentially more durable treatment option. Furthermore, the inclusion of sonographic measurement of tendon thickness provides objective evidence of the biological effects of PRP on tendon structure, complementing the subjective patient-reported outcomes.An important limitation of our ultrasonographic assessment is the focus solely on tendon thickness without evaluating other relevant parameters such as tendon echogenicity, fibrillar pattern, or neovascularity on power Doppler imaging. These additional features would provide a more comprehensive assessment of tendon healing. The reduction in tendon thickness observed in the PRP group suggests structural improvement but should not be interpreted as definitive evidence of tissue regeneration without histological or more detailed imaging confirmation.

Physiotherapy is widely recognized as a first-line treatment for supraspinatus tendinopathy. While our study did not directly compare PRP with physiotherapy, the literature suggests that PRP may serve as an adjunct or alternative for patients who do not respond adequately to physiotherapy. For example, Christopher found that PRP injections provided comparable outcomes to conservative treatments (which often include physiotherapy) in the treatment of partial rotator cuff tears [28].

Extracorporeal shockwave therapy (ESWT) is another conservative treatment option. Alian found that PRP injections led to better pain relief and functional improvement compared to ESWT at the 12-week follow-up for non-calcific supraspinatus tears [29]. Additionally, Moretti reported that a single PRP injection was non-inferior to ESWT in treating supraspinatus tendinosis, suggesting that PRP could be a viable alternative [30].

Our study has several limitations that should be acknowledged.The most significant limitation of our study is the absence of a placebo control group, which prevents us from definitively determining the absolute therapeutic effects of PRP versus the natural history of supraspinatus tendinopathy. As the condition can show spontaneous improvement in some patients, our results demonstrate relative superiority of PRP over corticosteroids but cannot quantify the absolute treatment effect. Second, the reliance on subjective outcome measures (VAS and Constant scores) may introduce bias, although these are standard tools in orthopedic research. Third, the study was not blinded due to the nature of the interventions, which could have influenced patient reporting and assessor bias.While the outcome assessor for our primary imaging endpoint was blinded, the study was not blinded for patients or the injecting physician due to the distinct nature of the two interventions. This could have introduced performance and detection bias, particularly for the subjective outcomes of VAS and Constant scores.Fourth, inter- and intra-observer reliability for the ultrasound measurements was not formally calculated in this study. However, we sought to minimize variability by having a single, highly experienced sonographer perform all baseline and follow-up measurements using a standardized protocol.A notable limitation is the co-administration of lidocaine in the corticosteroid group but not in the PRP group. This was done to adhere to standard clinical practice for each procedure but represents a potential confounder. The anesthetic effect of lidocaine likely contributed to the lower VAS scores observed in the corticosteroid group at the 1-week follow-up. However, as our primary finding relates to the superior efficacy of PRP at the 1-month and 3-month time points, long after any anesthetic effect would have subsided, we believe this initial bias does not alter the main conclusions of our study regarding the medium-term benefits of PRP.

Future research should aim to address these limitations and build upon our findings. There is a clear need for larger, multicenter, double-blind, randomized controlled trials with longer follow-up periods to confirm the long-term efficacy and safety of PRP for supraspinatus tendinopathy. Such studies should also include a placebo control group to account for the natural course of the condition and potential placebo effects. Moreover, given the established benefits of structured exercise programs for tendinopathies, future trials should investigate the potential synergistic effects of combining PRP injections with a standardized physical therapy protocol, particularly one that includes progressive load-control exercises [31]. This approach could potentially optimize patient outcomes by addressing both the biological and biomechanical aspects of the pathology. Finally, further research is needed to standardize PRP preparation protocols to ensure more consistent and comparable results across studies.

Future research should address these limitations by including a control group, incorporating objective imaging outcomes, and implementing blinding where possible. Additionally, longer follow-up periods are needed to assess the durability of treatment effects. Standardization of PRP preparation and administration protocols across studies would also facilitate more consistent comparisons.

Furthermore, direct comparisons between PRP and other conservative treatments, such as physiotherapy and ESWT, in randomized controlled trials would provide clearer insights into the relative efficacy of these interventions. Exploring the combination of PRP with rehabilitation programs could also be a promising avenue for enhancing treatment outcomes.

## Conclusion

Our study demonstrates that ultrasound-guided PRP injections show superior medium-term outcomes compared to corticosteroid injections for supraspinatus tendinopathy. However, the absence of a placebo control group limits our ability to determine the absolute therapeutic effect. These findings support PRP as a potentially superior alternative to corticosteroids, though further placebo-controlled trials are needed to establish definitive efficacy.

## Data Availability

Data and materials supporting the conclusions of this study are available from the corresponding author on reasonable request.
